# The Effect of Irreversible Electroporation on the Femur: Experimental Study in a Rabbit Model

**DOI:** 10.1038/srep18187

**Published:** 2015-12-10

**Authors:** Yue Song, Jingjing Zheng, Mingwei Yan, Weidong Ding, Kui Xu, Qingyu Fan, Zhao Li

**Affiliations:** 1 Department of Urologic and Pediatric Surgery, NO. 202 Hospital of People’s Liberation Army, NO. 5 Guangrong Street, Shenyang, 110003, P.R.China; 2 Orthopedics Oncology Institute of Chinese People’s Liberation Army and Department of Orthopedics, Tangdu Hospital, Fourth Military Medical University, NO. 1 Xinsi Road, Xi’an, 710038, P.R.China; 3 Department of Anesthesiology, The General Hospital of Shenyang Military Command, NO. 83 Wenhua Road, Shenyang, 110840, P.R.China; 4 Department of Electrical Engineering, Xi’an Jiaotong University, NO. 28 Xianning Road, Xi’an, 710049, P.R.China.

## Abstract

Irreversible electroporation (IRE) is a novel ablation method that has been tested in humans with lung, prostate, kidney, liver, lymph node and presacral cancers. As a new non-thermal treatment, the use of IRE to ablate tumors in the musculoskeletal system might reduce the incidence of fractures. We aimed to determine the ablation threshold of cortical bone and to evaluate the medium- and long-term healing process and mechanical properties of the femur in a rabbit model post-IRE ablation. The ablation threshold of cortical bone was between 1090 V/cm and 1310 V/cm (120 pulses). IRE-ablated femurs displayed no detectable fracture but did exhibit signs of recovery, including osteoblast regeneration, angiogenesis and bone remodeling. In the ablation area, revascularization appeared at 4 weeks post-IRE. Osteogenic activity peaked 8 weeks post-IRE and remained high at 12 weeks. The mechanical strength decreased briefly 4 weeks post-IRE but returned to normal levels within 8 weeks. Our experiment revealed that IRE ablation preserved the structural integrity of the bone cortex, and the ablated bone was able to regenerate rapidly. IRE may hold unique promise for *in situ* bone tissue ablation because rapid revascularization and active osteogenesis in the IRE ablation area are possible.

In 1992, Rosenthal *et al.* first described the use of radiofrequency ablation (RFA) to treat osteoid osteomas^1^. With improvements in imaging modalities, precise lesion localization and image-guided minimally invasive percutaneous tumor ablation have become increasingly common. RFA has replaced surgical resection for osteoid osteomas as the first-line treatment of choice because of its proven curative rates, shorter convalescence period, and decreased morbidity^2^,^3^,^4^. Palliation of painful metastatic bone disease with thermal ablation is considered safe and has been shown to reduce pain as well as improving the quality of life for cancer patients^5^,^6^. Given the success of these minimally invasive therapies, many orthopedic surgeons, oncologists, and interventional radiologists worldwide consider ablation techniques for the treatment and palliation of a variety of bone tumors^5^.

The most commonly used methods, including RFA, cryoablation, microwave ablation (MWA), high-intensity focused ultrasound, and interstitial laser photocoagulation, are hyperthermic methods. However, performing thermal ablation in long bones has been reported to potentially cause secondary fractures, particularly in the management of tumors involving weight-bearing bones, because the bone lesion causes weakening after tumor necrosis^2^,^7^. Thermal ablation can cause protein denaturation and coagulation necrosis^8^, and the damage from thermal ablation can induce long-term compensation by necrotic bone and elevate the risk for fracture.

Irreversible electroporation (IRE) is a novel ablation method that induces cell death by generating permanent membrane lysis or loss of homeostasis in the cell membrane using a short, high-voltage, direct electrical current^9^.The mechanisms of cell death after IRE are still not fully elucidated, and both necrosis and apoptosis are likely to occur^10^. IRE has been tested in humans with lung, prostate, kidney, liver, lymph node and presacral cancers^11^,^12^,^13^. Although IRE is believed to destroy all of the cells within the ablation zone effectively, the non-thermal nature of IRE results in relative preservation of the extracellular matrix^13^. The integrity of portal triad structures, the bowel wall, pancreatic duct, and urinary collecting system is protected because the collagen scaffold is retained, allowing regeneration^13^. As a result, the structural integrity of inlaying and adjacent tissue structures such as vessels, nerves and bile ducts remains intact^14^,^15^. As a new application, the use of IRE to ablate tumors of the musculoskeletal system might reduce the incidence of fractures. M. Fini *et al.* investigated the effect of IRE on the distal femoral epiphysis in a rabbit model and found that IRE induces the ablation of osteoblasts in mineralized trabeculae^16^. However, little is known about the medium- and long-term healing process and the mechanical properties of an ablated bone.

The purpose of this study was to determine the ablation threshold of cortical bone and evaluate the fate of the ablated bone segment by histopathological observations and mechanical competence assessments.

## Results

### Clinical Observations

Of the 165 animals used in our experiment, 4 animals died from anesthetic accidents, and another 3 animals died from deep infection after surgery; all of the vacancies were filled accordingly. All of the other animals survived the procedures without complications.

### Distribution of the Electric Field Intensity and Determination of the Effective Ablation Threshold

**Figure 1:**
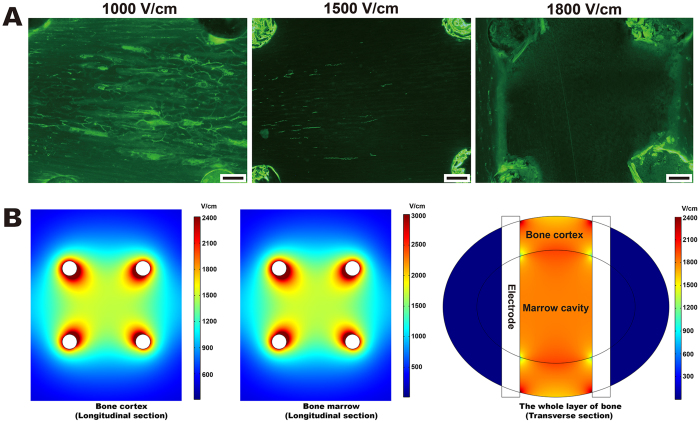
Determination of the effective ablation threshold. (**A**) Effectiveness of IRE ablation using three different electrical intensities. One hundred twenty direct-current pulses at 1,000 V/cm, 1,500 V/cm or 1,800 V/cm were applied to animals in the threshold group. Calcein green labeling was observed 1 week after IRE ablation at 1,000 V/cm and at 1,500 V/cm, but no labeling was observed at 1,800 V/cm. Bars represent 500 μm. (**B**) Distribution of the electric field intensity at 1,800 V/cm. Pulses of 1,800 V/cm were output 6 times, including on four sides and two diagonals of the square configuration. The electric field distribution in a square configuration was non-uniform, as evidenced by the graduated color. In the longitudinal section, the lowest intensity occurred at the center of the square (1,610 V/cm in the cortex and 1,730 V/cm in the marrow). In the transverse section, the lowest intensity occurred at the intersection of the cortex, marrow and electrode (1,310 V/cm).

When 1,000 V/cm or 1,500 V/cm was applied to the bone tissue, calcein green fluorescence was observed on the cortex seven days after IRE (Fig. 1A). However, no fluorescence labeling was observed inside the ablation unit under a stimulus of 1,800 V/cm. An effective ablation parameter of 120 pulses at 1,800 V/cm was thus confirmed and applied to the animals in the IRE groups. According to the modeling of the electrical field, under a stimulus of 1,800 V/cm, the electric field distribution in a geometric square was non-uniform (Fig. 1B). The lowest intensity occurred at the center of the square (1,610 V/cm in the cortex and 1,730 V/cm in the marrow) in the longitudinal section, whereas the lowest intensity occurred at the intersection of the cortex, marrow and electrode (1,310 V/cm) in the transverse section. Likewise, when applying 1,500 V/cm, the lowest intensity in the bone was 1,090 V/cm. Therefore, assuming 1,800 V/cm can effectively ablate all of the bone tissues surrounded by the four electrode holes, we theorized that the effective threshold for bone cortex ablation is between 1,090 V/cm and 1,310 V/cm (120 pulses).

### Histopathological Assessment

**Figure 2:**
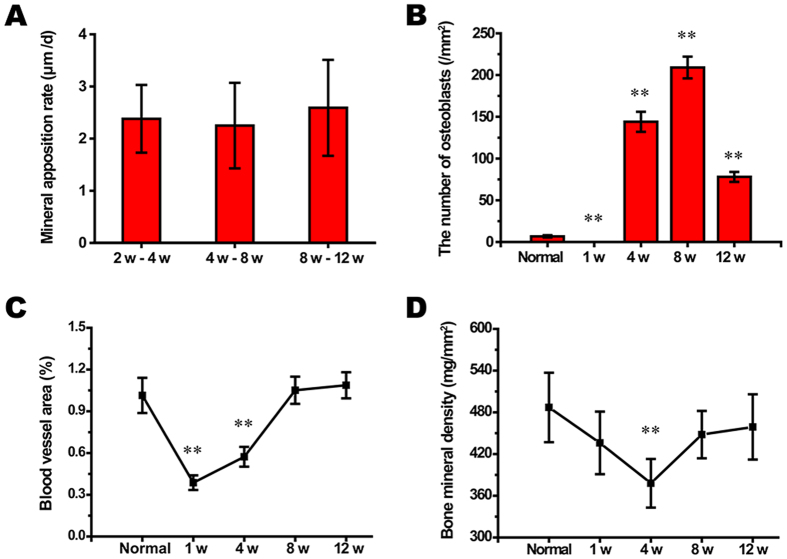
Statistical evaluation of the morphological observation and determination of bone mineral density. (**A–D**): *n* = 12, **P < 0.001 compared to the normal value. Variables in each group were fitted or approximated to the normal distribution. Homogeneity of variance test for variables in each group showed *P* > 0.05. The “week” was abbreviated as “w”.

**Figure 3:**
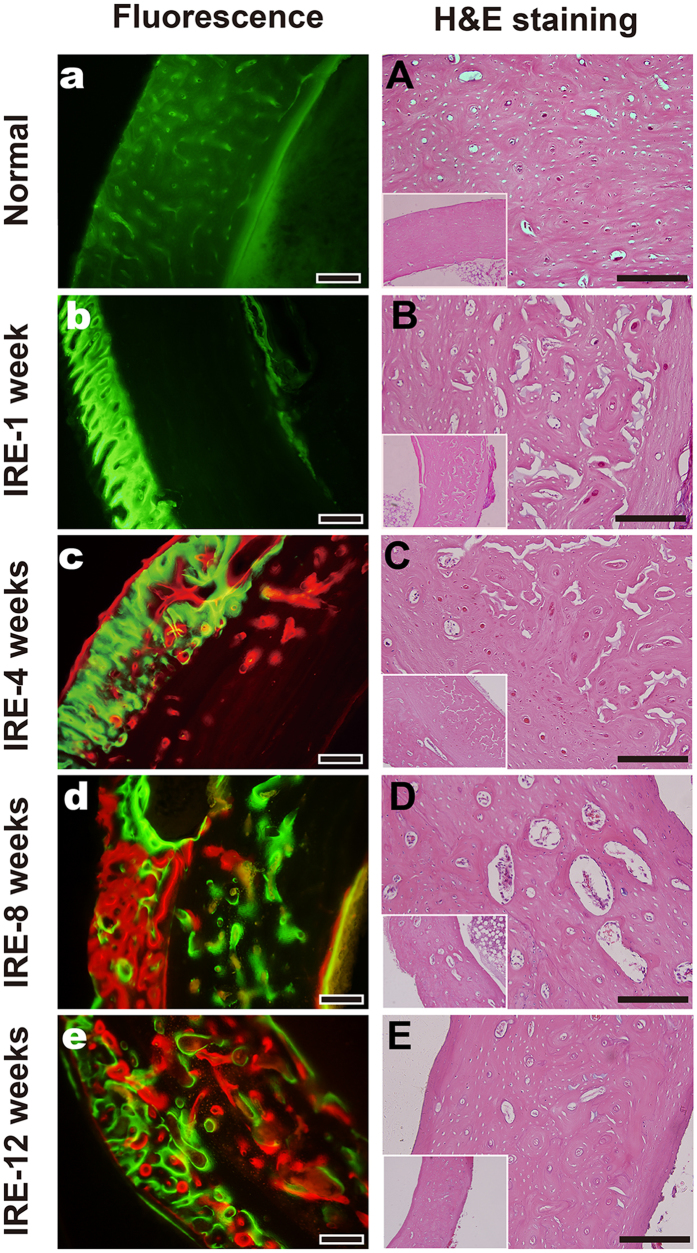
Histopathological assessment of the IRE-ablated bone with fluorescence and H&E staining. (**a–e**): Fluorescent labeling of the target bone segment with calcein green or alizarin red in the corresponding period. (**A–E**): H&E staining of the target bone segment in the corresponding time period. Bars represent 200 μm.

**Figure 4:**
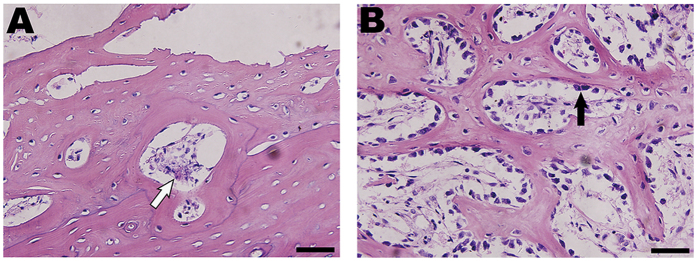
Histopathological assessment of the IRE-ablated bone cortex and trabecula at 4 weeks with H&E staining. (**A**) Bone cortex: osteoclasts (white arrow) excavated a resorption cavity known as a cutting cone and formed a linear resorption tunnel. (**B**) Bone marrow: osteoblasts (black arrow), appearing cuboidal, lined up at the rims of the trabeculae. Bars represent 50 μm.

During the entire 12-week observation period, three consecutive time periods of 2–4 weeks, 4–8 weeks and 8–12 weeks were chosen to compare changes in the mineral apposition rates (MARs) according to the mean width between double fluorescence labeling. There were no differences in the MARs between the three time periods (Fig. 2A), although the number of osteoblasts per area showed significant changes (*n* = 12, **P < 0.001 compared to the normal value, Fig. 2B). In normal bone specimens, the deposition of new bone was evident in the cortex and marrow (Fig. 3a), and the bone structural units (BSUs) were regularly distributed within the bone cortex (Fig. 3A). At 1 week, the fluorescent labeling on the bone cortex was blocked due to the complete ablation of osteocytes, but a thin layer of callus formed at both the outer and inner periosteum surfaces, which facilitated bone regeneration (Fig. 3b). Bone resorption appeared evident in the outer layer of the bone cortex, where osteoclasts resorbed the bone matrix, leaving the remaining lacuna *in situ* (Fig. 3B). At 4 weeks, new bone formation marked by calcium deposition was evident in the outer layer of the bone cortex (Fig. 3c). Osteogenic activity and bone resorption coexisted, and resorption of the ablated bone tissue was still occurring, particularly in the inner layer (Fig. 3C). Osteoclasts excavated a resorption cavity known as a cutting cone, and formed a linear resorption tunnel (Fig. 4A). Osteoblasts with a cuboidal morphology lined up at the rims of trabeculae and the inner surface of the BSU (Fig. 4B). At 8 weeks, the osteogenic activity was more evident over the full thickness of the bone cortex, exhibited by the area of bone mineralization (Fig. 3d). Some osteoblasts were flattened or entrapped in the osteoid and ultimately became osteocytes. Bone resorption was complete at 8 weeks (Fig. 3D). At 12 weeks, the callus thinned or even disappeared, and the number of osteoblasts declined. Newly formed BSUs were irregularly distributed throughout the full thickness of the bone cortex, indicating ongoing bone remodeling (Fig. 3e,E).

### Angiogenesis Analysis

**Figure 5:**
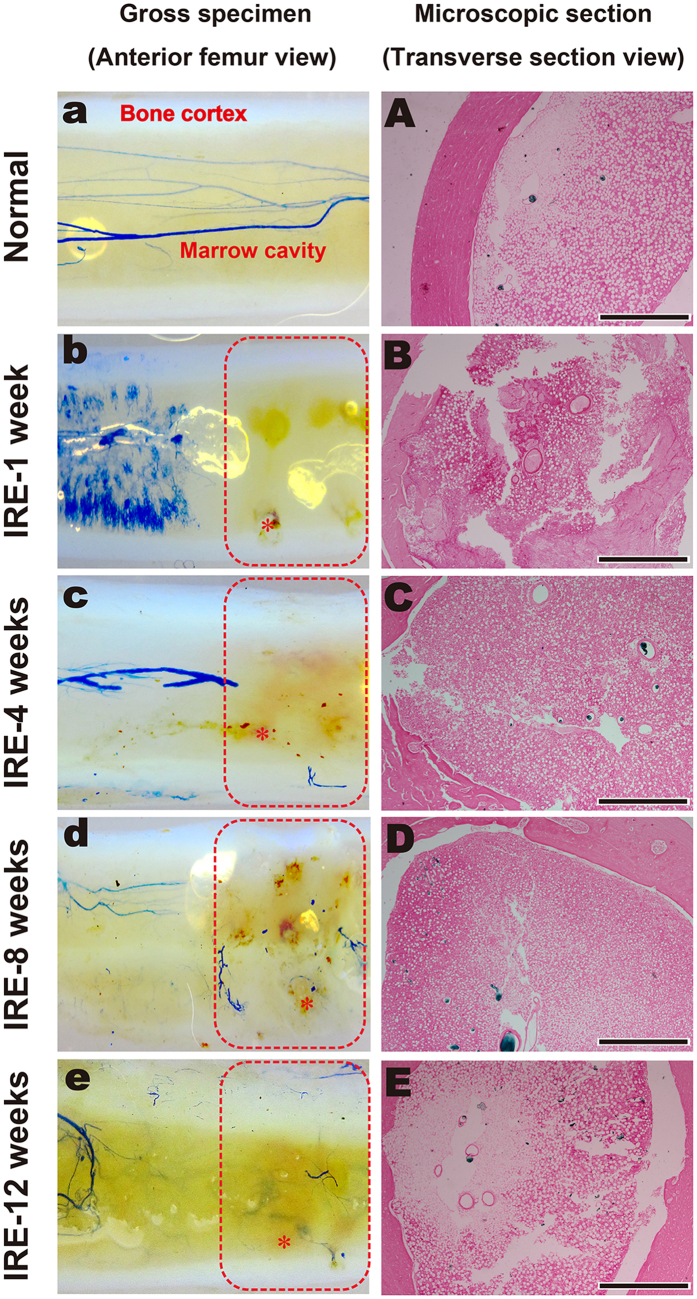
Angiogenic analysis by gross observation and microscopic examination. (**a–e**) In the gross observation, the ablation area was surrounded by red dotted borders; one of the electric holes in an image is marked by “*”. (**A–E**) The microscopic section is within the ablation area. IRE-1 week: Vascular perfusion was not observed by gross observation (**b**). Complete occlusion of the vessel lumen occurred without blood vessel wall damage microscopically (**B**). IRE-4 weeks: Revascularization was scattered throughout the full ablation area (**c**). The blood vessel cavities were intact, although some were not filled with Microfil (**C**). IRE-8 weeks: The blood vessels in the ablated area gradually became coherent overall (d) and received a sufficient blood supply microscopically (**D**). IRE-12 weeks: The continuity of blood vessels was almost consistent with that of the normal bone group (**e,E**). Bars represent 500 μm.

Complete occlusion of the vessel lumen occurred 1 week post-IRE, when vascular perfusion was not visible in the gross specimens (Fig. 5b). The blood cell necrosis, local edema and high pressure in the bone marrow cavity might have acted together to cause a temporary loss of blood supply to the ablation segment (Fig. 2C). Although the vascular lumens were full of thromboses, the blood vessel walls were intact (Fig. 5B). The blood supply might not have been blocked permanently, as revascularization was observed at 4 weeks. Although limited and scattered, the necrotic trabeculae and endosteal bone were being replaced by new bone (Figs 5c and 2C). At this time point, some of the blood vessel cavities were not filled with Microfil, but the vessel walls were intact, demonstrating an ability to deliver blood (Fig. 5C). At 8 weeks, revascularization was clearly apparent; moreover, the blood vessels in the ablated segment gradually became coherent and were sufficient to provide a blood supply (Figs 5d,D and 2C). At 12 weeks, the continuity of blood vessels in the IRE group tended to be equal to that of the normal bone group (Fig. 5e,E).

### X-ray Observation and Bone Densitometry

**Figure 6:**
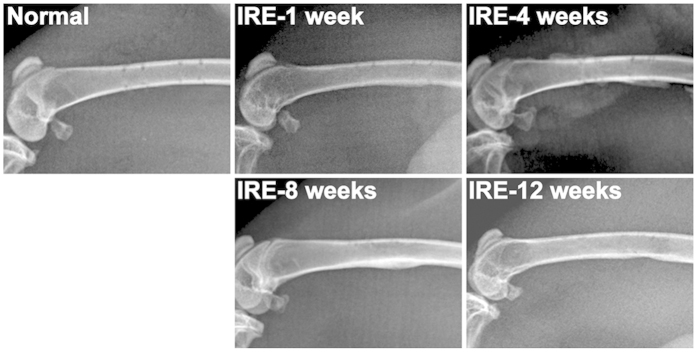
X-ray observation of the IRE-ablated bone in the corresponding period. Normal: The electrode holes were visible. IRE-1 week: A fibrous callus packed the holes, and a thin layer of calcified callus formed in the posterior of the femur. IRE-4 weeks: The callus in the outer layer of the cortex thickened, and a new callus filled the electrode holes. IRE-8 weeks: The callus thinned. IRE-12 weeks: The callus almost disappeared.

The electrode holes were visible in the normal bone specimen (Fig. 6). At 1 week, fibrous callus packed the holes, and a thin layer of calcified callus formed in the posterior of the femur. At 4 weeks, the callus in the outer layer of the cortex thickened, and new trabecular bones filled the electrode holes. The callus thinned at 8 weeks and almost disappeared by 12 weeks. Bone densitometry indicated that the bone mineral density value at 4 weeks significantly decreased to 378 ± 35 mg/mm^2^ (*n* = 12, **P = 0.007 compared to the normal value) but returned to normal at 8 weeks (Fig. 2D).

### Biomechanical Test

**Figure 7:**
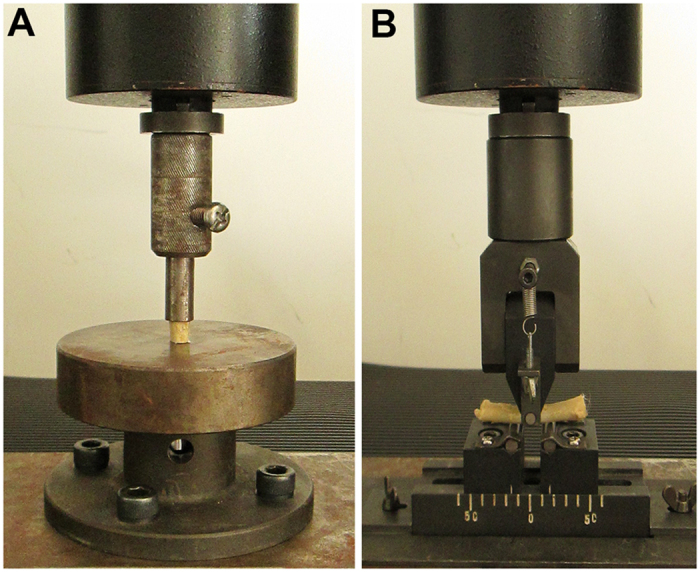
Biomechanical testing of the IRE-ablated bone segment. (**A**): Compression test. (**B**): Bending test.

Mechanical tests were performed as depicted in Fig. 7. Table 1 lists the biomechanical parameters of the bone specimens. The mechanical strength of the specimens at 4 weeks decreased significantly (*n* = 12, **P < 0.001 compared to the normal value). In the compression test, the maximum load and energy absorption at 4 weeks were 874 ± 85 N and 333 ± 32 mJ, which were approximately 53% and 49% of the values in the normal bone group, respectively. No differences in deformation or stiffness were observed between all of the groups. In the bending test, the maximum deformation and stiffness at 4 weeks were 1.78 ± 0.13 mm and 476 ± 81 N/mm (*n* = 12, **P < 0.001 compared to the normal value), respectively, which were approximately 231% and 50% of the values in the normal bone group. There were no differences in the maximum load or energy absorption between all of the groups.

## Discussion

Our experiment revealed that IRE ablation spared the structural integrity of the bone cortex and that the ablated bone was able to regenerate rapidly. Although IRE ablation had negative effects on bone mechanics at 4 weeks, these effects did not cause fracture and abated within 8 weeks.

For homogeneous tissue, the distribution of an electric field can easily be predicted^17^. Bone, however, is highly heterogeneous. The electric fields in bone are non-uniform, especially at the border of the cortex and the marrow. The literature contains little information regarding the IRE threshold for bone tissue. M. Fini *et al.* identified the effective ablation threshold as 3,500 J/kg^16^; however, in most studies, the electric field strength is considered to be the ablation threshold. For example, 1800 V/cm with ninety 100-μs pulses was used for rabbit Achilles tendon^18^, and 1000 V/cm with one hundred 100-μs pulses was used for porcine liver^19^. In our experiments, the bone cortex threshold was determined to be between 1,090 V/cm and 1,310 V/cm (120 pulses), which were significantly higher than for previously characterized tissues. One possible reason for this difference is that the osteocyte lies within a lacuna.

Bone regeneration is generally considered a slow process. Foster, L. N. *et al.* studied cortical infarcts in the bones of rabbits and found that large amounts of dead bone cortex were present after 9 months^20^. Yamamoto, S. *et al.* evaluated the heat effects of RFA on the normal bones of rabbits. They concluded that heat induced by RFA did not change the normal bone strength within 2 months^21^. It is difficult to believe that the death of osteocytes would not affect bone strength. Ji, Z. *et al.* assessed a devitalized bone segment using a dog model. Serious bone resorption was observed 2 months after surgery, and 16.67% of the dogs developed fractures. At 1 month, only certain areas adjacent to the endosteum were revascularized. In our study, blood flow was present in the marrow at 4 weeks and had nearly returned to normal levels 8 weeks post ablation; moreover, new bone formation was observed after 8 weeks over the full thickness of the cortex. *In situ* bone regeneration after IRE appears to occur more rapidly than regeneration following other ablation methods.

Several unique characteristics distinguish IRE from other current tumor ablative techniques.

1. Unlike thermal ablation methods, IRE is not associated with a temperature increase or with protein denaturation if not in a sufficiently strong field^22^; rather, IRE effectively preserves the protein activity^23^. Osteoconduction, osteoinduction, and osteoblasts are three required factors for bone regeneration^24^. Growth factors are usually stored in the extracellular matrix of the bone and play an osteoinductive role during bone regeneration^25^. The state of the bone cortex following IRE might preserve osteoconduction and osteoinduction, whereas heated bone only preserves osteoconductive ability.
2. IRE causes complete tissue death through apoptosis or “apoptosis-mimetic” necrosis^26^, which has many beneficial effects. For instance, there is emerging evidence that apoptotic cells promote tissue regeneration. The apoptosis of osteocytes instructs neighboring viable osteocytes to synthesize cytokines such as RANKL and VEGF, which recruit osteoclasts to remove the dead cells and initiate the remodeling of the surrounding matrix^27^. This process enables the extremely rapid regeneration of ablated tissue^28^.
3. IRE might have a unique ability to spare critical structures^9^,^13^,^22^. We observed the preservation of complete blood vessel walls and rapid recovery of the blood supply. The extraordinarily rapid rate of bone regeneration in our experiment suggests that bone regeneration is not restricted to the interface of normal bone and dead bone. The vascular contribution is fundamental for both necrotic tissue resorption and osteogenesis. Vessels represent a vehicle for mesenchymal cells that consequently begin to proliferate and differentiate in the osteoclastic and osteoblastic pathways^29^. Consequently, bone regeneration might be possible in every area that is revascularized. Of course, further experiments are necessary to confirm this hypothesis.

Effective maintenance of overall tissue integrity and reduced fibrosis have been reported as favorable side-effects of non-thermal IRE in comparison to other thermal ablation methods^30^. Traditional hyperthermic methods can cause coagulative necrosis of both the tumor and adjacent tissues^31^. Osteocyte death can compromise bone strength^32^. The degeneration of collagen causes bone fragility and carries a risk of secondary bone fracture^33^,^34^. Indeed, the load for normal physiological activity is far less than the load for fracture. The rabbits in our study were fully weight bearing, and no occurrences of fracture were observed. The mechanics experiment demonstrated that the basic mechanical strength of the femur was preserved. Thus, IRE could theoretically be a promising option for treating bone tissues without causing secondary fractures.

When IRE is used alone for cell ablation, the electrical field delivered is relatively high. Some authors suggest that IRE should be used in combination with chemotherapy^16^,^30^. IRE allows increased uptake of chemotherapeutic drugs into tumor cells^22^. When electrochemotherapy (ECT) has been used, the electrical field threshold does not need to exceed the primary standard, and the number of pulses delivered can also be limited^35^. With ECT, the combined effect of IRE and cytotoxic drugs can be particularly effective, as it allows large volumes of tumor to be treated^36^.

Nevertheless, certain limitations of this study must be acknowledged. First, the limited length of ablation might limit the clinical generalizability when ablating a larger bone segment. Second, this investigation was limited by the relatively short period of follow-up observation. Third, electric field simulation should be further optimized because the unique and heterogeneous properties of bone tissue might cause unpredicted ablation effects^37^. Fourth, electrical conductivity changes due to electroporation should be incorporated in future studies. Fifth, additional immunohistochemistry is needed to identify cell types during regeneration time. Sixth, further studies using a bone tumor model and comparing IRE to other methods are necessary to better evaluate IRE for treating bone tumors.

In conclusion, IRE performs effectively in normal bone ablation. Because of advantages such as the possibility of rapid revascularization and osteogenic activity in the ablated area, IRE might be a promising method for managing a variety of bone tumors.

## Methods

### Animals and Ethics Statement

One hundred sixty-five male, 6-month-old New Zealand rabbits with an average weight of 2.6 ± 0.5 kg were randomly divided into three groups: 15 for the ablation threshold determination, 30 for the sham operation (normal bone group), and 120 for electroporation ablation (IRE group). The animals in the IRE group were further randomly divided into four sub-groups (30 animals per group) by euthanization time: 1 week, 4 weeks, 8 weeks or 12 weeks. The animals were divided within each sub-group as follows: 6 for histological observation, 6 for fluorochrome labeling, 6 for microvascular perfusion and 12 for biomechanical testing. In all of the experiments, the bilateral femurs of an experimental rabbit were treated as specimens. In the biomechanical test, the left femur in a rabbit was ablated for the compression test, and the right one for the bending test, respectively. All of the experimental procedures involving animals were performed under a protocol that was reviewed and approved by the Ethics Committee of Tangdu Hospital, Fourth Military Medical University (Permit number: TDLL2012034). All of the animal work was conducted in accordance with national and international guidelines to minimize animal suffering.

### Surgical Procedure and Tissue Ablation

The animals were anesthetized with an intramuscular injection of diazepam (300 μg/kg) and xylazine (5 mg/kg).

A square configuration of 4.9 mm spacing for each side (the diameter of an electrode plus the edge-to-edge distance between adjacent electrodes) was set as an ablation unit, and its four corners were set as electrode holes. Two stainless steel electrodes, 0.9 mm in diameter, were inserted into double cortexes and marrow in the same side or the same diagonal in a square. We delivered 12 trains of 10 direct current square pulses per electrode couple at 1,000 V/cm, 1,500 V/cm or 1,800 V/cm with a pulse duration of 100 microseconds, a pulse interval of 100 milliseconds and a train interval of 2 seconds. Pulses were outputted from a pulse generator (TP3032, Teslaman, Dalian, China) and applied to all of the six potential electrode pairs. The voltage applied was expressed in V/mm, and the actual electric field applied to the electrodes was adjusted for electrode separation. For example, the electric field applied between electrodes was 720 V/4.00 mm and 1,085 V/6.03 mm (edge-to-edge distance on a diagonal). This conversion yielded a nominal electric field of 1800 V/cm. Animals in the IRE groups were then subjected to 120 pulses at 1,800 V/cm, and animals in the normal bone group were subjected to no pulse output. During the first 24 hours after surgery, the animals were given two doses of meloxicam (2 mg/kg) at 8-hour intervals. All of the animals were checked daily to ensure that they recovered, remained healthy, and were not experiencing pain.

### Modeling of the Electrical Field

The distribution of the electrical field in the femur was calculated using COMSOL software (Multiphysics, Massachusetts, U.S.). The conductivity values were 4.0e^6^ [S/m] for the electrode, 1e^−17^ [S/m] for the insulating layer, 2.04e^−2^ [S/m] for the bone cortex and 3.23e^−3^ [S/m] for the bone marrow. The dielectric constants were 1 for the electrode, 4.5 for the insulating layer, 522 for the bone cortex and 676 for the bone marrow. The conductivity and dielectric constant of the bone cortex and marrow were measured using a signal analyzer (N9030A PXA, Agilent, U.S.).

### Histological Examination

Whenever bone matrix mineralizes, new bone formation (including both the cortex and the marrow) can be labeled with fluorochromes such as calcein green (10 mg/kg) or alizarin red (30 mg/kg). Fluorochromes were subcutaneously injected once or twice as follows: 1-week group: calcein green (on the 5th day post-ablation, ‘5th day’ for short); 4-week group: calcein green (12th day) and alizarin red (26th day); 8-week group: alizarin red (26th day) and calcein green (54th day); 12-week group: alizarin red (54th day) and calcein green (82nd day)^38^. At each sampling time point, the specimens were fixed in 4% formalin for 24 hours. Twelve specimens were then decalcified, embedded in paraffin, and sectioned transversely for H&E staining. Another 12 specimens were dehydrated in a graded series of alcohols, embedded in polymethylmethacrylate, and sectioned transversely using a Leica 1600 diamond saw microtome for fluorescent observation. The double fluorescence labeling width was measured using Image-Pro Plus (version 6.0 Media Cybernetics, Inc., Bethesda, MD, U.S.). MAR was calculated as the interlabel width/interval (μm/day)^39^.

### Microvascular Perfusion and Microvessel Quantitation

The animals were anesthetized, cannulated in the abdominal aorta, and heparinized with physiological saline solution (12,500 IU/L) in advance. Following euthanization, 50 mL of blue polymerizing contrast compound (Microfil^®^; Flow Tech Inc., U.S.), which is designed for filling blood vessels greater than 10 microns, was injected under physiological pressure^40^,^41^. The cannulated segments were first decalcified, dehydrated and embedded in methyl salicylate for gross observation and then were embedded in paraffin and cut into transverse sections for microscopic observation. Eosin staining was used to distinguish bone tissue from blood vessels. Microvascular quantification was defined as the ratio of the vessel (Microfil^®^) pixels to the total bone pixels using Image-Pro Plus.

### X-Ray Observation and Bone Mineral Density Measurement

Each of the femurs used in the compression tests was X-rayed before sampling. Each of the femurs used in the bending tests was dual energy X-rayed before biomechanical testing. Bone mineral density measurements were performed using J-Vision software (Agfa, Mortsel, Belgium).

### Biomechanical Examination

The femoral samples were cut to 10 mm and 40 mm in length for the compression test and three-point bending test, respectively (with 12 specimens in each test). The compressive strength was applied at a speed of 1 mm per minute for each test, and the load deflection curve was recorded simultaneously.

### Statistical Analysis

Data are presented as the mean value ± standard deviation (mean ± SD). A numerical method using skewness and kurtosis was selected to examine normality. A normally distributed random variable should exhibit skewness and kurtosis near zero and three, respectively. Whenever the variables follow normal probability distributions, one-way analysis of variance was used to evaluate differences among groups. The Levene test was used for the homogeneity-of-variance analysis, in which the “alpha” level was set at 0.05. The Bonferroni test was used for multiple comparisons. Differences between the means were considered significantly different when two-sided *P* values were less than 0.05. All of the statistical analyses were performed using SPSS software (version 17.0, SPSS, Inc., Chicago, IL, U.S.).

## Additional Information

**How to cite this article**: Song, Y. *et al.* The Effect of Irreversible Electroporation on the Femur: Experimental Study in a Rabbit Model. *Sci. Rep.* **5**, 18187; doi: 10.1038/srep18187 (2015).

## Figures and Tables

**Table 1:** Mechanical parameters in the compression and bending tests.

Groups	Compression test				Bending test			
	Maximum load (N)	Maximum deformation (mm)	Stiffness (N/mm)	Energy absorption (mJ)	Maximum load (N)	Maximum deformation (mm)	Stiffness (N/mm)	Energy absorption (mJ)
Normal	1653 ± 180	0.85 ± 0.12	2421 ± 368	681 ± 55	292 ± 45	0.77 ± 0.13	985 ± 125	83 ± 11
1 week	1455 ± 88	0.92 ± 0.16	2656 ± 296	599 ± 52	255 ± 59	1.03 ± 0.20	865 ± 108	91 ± 23
4 weeks	874 ± 85**	1.02 ± 0.15	2161 ± 323	333 ± 32**	268 ± 72	1.78 ± 0.13**	476 ± 81**	106 ± 22
8 weeks	1327 ± 157	0.81 ± 0.12	2063 ± 470	751 ± 82	347 ± 57	0.94 ± 0.17	1089 ± 135	97 ± 19
12 weeks	1468 ± 69	1.09 ± 0.20	2693 ± 310	792 ± 34	360 ± 86	0.96 ± 0.12	1099 ± 127	94 ± 21

Mean (SD): **Significant difference in the corresponding period (**P < 0.001 compared to the normal value, respectively, *n* = 12).
